# Desipramine restores the alterations in circadian entrainment induced by prenatal exposure to glucocorticoids

**DOI:** 10.1038/s41398-019-0594-3

**Published:** 2019-10-17

**Authors:** Stefan Spulber, Mirko Conti, Frederik Elberling, Marilena Raciti, Dasiel Oscar Borroto-Escuela, Kjell Fuxe, Sandra Ceccatelli

**Affiliations:** Department of Neuroscience, Karolinska Institutet, Stockholm, Sweden

**Keywords:** Depression, Molecular neuroscience

## Abstract

Alterations in circadian rhythms are closely linked to depression, and we have shown earlier that progressive alterations in circadian entrainment precede the onset of depression in mice exposed in utero to excess glucocorticoids. The aim of this study was to investigate whether treatment with the noradrenaline reuptake inhibitor desipramine (DMI) could restore the alterations in circadian entrainment and prevent the onset of depression-like behavior. C57Bl/6 mice were exposed to dexamethasone (DEX—synthetic glucocorticoid analog, 0.05 mg/kg/day) between gestational day 14 and delivery. Male offspring aged 6 months (mo) were treated with DMI (10 mg/kg/day in drinking water) for at least 21 days before behavioral testing. We recorded spontaneous activity using the TraffiCage™ system and found that DEX mice re-entrained faster than controls after an abrupt advance in light-dark cycle by 6 h, while DMI treatment significantly delayed re-entrainment. Next we assessed the synchronization of peripheral oscillators with the central clock (located in the suprachiasmatic nucleus—SCN), as well as the mechanisms required for entrainment. We found that photic entrainment of the SCN was apparently preserved in DEX mice, but the expression of clock genes in the hippocampus was not synchronized with the light-dark cycle. This was associated with downregulated mRNA expression for arginine vasopressin (AVP; the main molecular output entraining peripheral clocks) in the SCN, and for glucocorticoid receptor (GR; required for the negative feedback loop regulating glucocorticoid secretion) in the hippocampus. DMI treatment restored the mRNA expression of AVP in the SCN and enhanced GR-mediated signaling by upregulating GR expression and nuclear translocation in the hippocampus. Furthermore, DMI treatment at 6 mo prevented the onset of depression-like behavior and the associated alterations in neurogenesis in 12-mo-old DEX mice. Taken together, our data indicate that DMI treatment enhances GR-mediated signaling and restores the synchronization of peripheral clocks with the SCN and support the hypothesis that altered circadian entrainment is a modifiable risk factor for depression.

## Introduction

Alterations in circadian rhythms are closely linked to psychiatric conditions, and disruption of rest/activity patterns in relation to the 24-h cycle are typically present in depressed patients^1,2^. Interfering with circadian rhythms, such as by shift work for extended periods of time, or with circadian clues (e.g., artificial light at night), increases the risk of developing depression^3–5^, while therapies aiming at restoring circadian rhythms (e.g., light therapy, behavioral activation) are effective in mild-to-moderate depression^6^. In animal models, depression-like behavior can be induced by chronic light deprivation^7^, chronic exposure to light^8,9^, or even direct interference with the function of the central clock^10^.

The ability to adapt to environmental changes is a fundamental feature of all living organisms and predictive adaptation to regularly recurring environmental challenges is made possible by the presence of molecular clocks in virtually all cells in the mammalian organism^11^. The core molecular clock machinery consists of a set of transcription factors (clock genes) engaged in interlocked feedback loops which generate self-sustained oscillations with a period ~24 h (i.e., circadian oscillations)^12,13^. In addition, the molecular clock machinery has a degree of flexibility which allows the synchronization (entrainment) with an extrinsic periodic stimulus (pacemaker, e.g., pulsatile hormone secretion)^12,13^. The central clock is located in the anterior hypothalamus in the suprachiasmatic nucleus (SCN) and consists of several neuronal populations that display prominent spontaneous cyclic fluctuations in firing patterns and synchronized circadian oscillations in clock gene expression^13^. Importantly, the oscillation in clock gene expression and neuronal activity in the SCN maintain synchronization even without external entrainment input^14,15^. In contrast, peripheral clocks are cell populations which express functional clock machinery, but require constant entrainment by the central clock^11^. The hypothalamic-pituitary-adrenal (HPA) axis plays a critical role in the hierarchical organization of molecular clocks^16–18^. Information on environmental light (the main circadian entrainer) reaches the SCN via the retinohypothalamic tract (RHT) and synchronizes the central clock with the light-dark (LD) cycle (photic entrainment). The SCN regulates the activity of HPA axis by means of arginine-vasopressin (AVP) signaling in the paraventricular hypothalamic nucleus^18,19^. Peripheral clocks are then entrained through rhythmic release of glucocorticoids (GC)^20–23^. The core function of the SCN is to synchronize biological rhythms with the LD cycle, and requires flexibility to adapt to changes in the environment, but also entails resistance to sudden changes^11,13^.

In utero exposure to the synthetic GC analog dexamethasone (DEX) is an established model to induce intrauterine growth retardation (IUGR)^24,25^, a condition associated with detrimental consequences in humans, including neuropsychiatric disorders, such as depression^26^. Using this model we have shown that mice display alterations in photic entrainment of spontaneous activity which precede the onset of depression-like behavior^27^. Briefly, we found that mice exposed to DEX display rigid patterns of activity under constant LD cycle (steady photic entrainment), and faster re-entrainment to a phase-advanced LD cycle^27^. In addition, the amplitude of circadian oscillations in GC is lower than in controls, suggesting weaker entrainment of peripheral clocks. We hypothesized that uncoupling peripheral clocks from the SCN is a mechanism underlying the late-onset depression-like behavior^27^. The aim of this study was to investigate whether the alterations in photic entrainment can be reversed by restoring the coupling between central and peripheral clocks before the onset of depression. To this end we have treated adult mice exposed to DEX in utero with desipramine (DMI), a tricyclic antidepressant blocking preferentially norepinephrine (NE) reuptake^28^, which has been shown to enhance GC signaling^29–32^. We found that DMI treatment of DEX-exposed mice delayed the re-entrainment of spontaneous activity and restored the synchronization of hippocampal clock gene expression with the LD cycle. In addition, DMI treatment at 6 months of age prevented the increase in immobility time and the alterations in hippocampal neurogenesis at 12 months.

## Materials and methods

### Animals and treatments

All experiments were conducted in agreement with the European legislation (2010/63/EU Directive) and Swedish national regulation following approval by the local Animal Ethics Committee (Stockholms Norra djurförsöksetiska nämnd).

DEX (Sigma-Aldrich, Stockholm, Sweden) was dissolved in sterile saline to a concentration of 5 µg/mL. Pregnant C57BI/6 dams (*N* = 15 females/group) (Charles River, SCANBUR Research, Sollentuna, Sweden) received a subcutaneous injection of 0.05 mg/kg/day DEX or equivalent volume of vehicle (10 mL/kg b.w. physiological saline), from gestational day 14 until delivery. The litters were culled to 4 pups per litter at post-natal day (PND) 3 and weaned at PND21. At the time of weaning, the animals were distributed in groups of 4–5 mice per cage so that each mouse originated in a different litter, and were tagged with subcutaneous radio frequency identification (RFID) transponders (Trovan™ Unique T-100A, Trovan, UK) under brief 4% isoflurane anesthesia. The RFID tags allowed unambiguous identification of individual animals throughout the experiment. Only the male pups were used for subsequent experiments, since female offspring did not show similar behavioral alterations (see also ref. ^27^). The timeline of experiments is shown in Supplementary Fig. S1.

Mice aged 5 months (mo) were treated with DMI (Sigma-Aldrich, Stockholm, Sweden) dissolved in drinking water (10 mg/kg/day) for 28 days before recording spontaneous activity, and the treatment was continued throughout the recording period. We did not observe changes in fluid intake after adding DMI to drinking water (not shown).

### Recording and analysis of spontaneous locomotor activity in the homecage

All experiments involving circadian rhythms were performed in an isolated room (22 ± 1 °C; 50 ± 5% relative humidity), with 12:12 h LD cycle (light intensity 200 lx) and free access to food and water. The circadian zeitgeber (German: “time-giver”) time (ZT) 0 corresponds to the onset of the light phase. The interaction with human experimenters was limited to changing the cage and replenishing food and water, which occurred at random times throughout the experiment. The mice were kept in conditions similar to the environment in the holding facility (i.e., group-housed in the homecage as assigned at weaning), except the top of the cage was kept clear to ensure even illumination of the cage bottom.

We recorded the spontaneous activity of mice (*N* = 7–8/group) using the TraffiCage™ system (NewBehavior™, Zürich, Switzerland), as described elsewhere^27^. The system consists of an array of antennas placed under the cage. The antennas read the RFID tags and provide the location of each animal with a time resolution of 20 ms. A “visit” was defined as the time interval during which an animal is detected constantly by the same antenna, and was used as activity count. Each recording session included two cages monitored simultaneously, one control and one test cage, placed randomly on either TraffiCage™ plates. After reaching stable circadian entrainment to the 12:12 h LD cycle, the light was turned off 6 h earlier (advance the onset of dark phase) to induce a phase advance in the circadian rhythms of activity. The activity was monitored for at least four LD cycles before, and at least five LD cycles after the phase shift.

### Detection of onset of active phase for assessment of photic entrainment

The time series of activity counts were exported as ASCII files and analyzed using custom algorithm implementations in Matlab™ 9 (The MathWorks™, Natick, MD, USA). To identify the onset of active phase, the activity was binned in 5 min non-overlapping epochs, and smoothed by weighted average with a sliding Gauss window (2 h width). The epochs with activity above the individual’s detrended average were considered “active epochs”. The active phase was defined as a sequence of active epochs contiguous or separated by gaps no larger than 1 h. The onset of active phase was defined as the time corresponding to the beginning of the active phase (relative to subjective time, ZT; ZT0 = light on). We defined spontaneous activity as entrained to the LD cycle if the onset of the active phase occurred within 1 h from the onset of the dark phase. The individual time to re-entrain was estimated as the first day when spontaneous activity was entrained to the shifted LD cycle.

### Analysis of periodicity and variability in spontaneous activity

The periodicity of spontaneous activity was analyzed using the Lomb-Scargle periodogram^33^ in non-overlapping contiguous 24 h epochs using a publicly available implementation of the algorithm in Matlab™ (https://se.mathworks.com/matlabcentral/fileexchange/20004-lomb–lomb-scargle–periodogram). The false alarm probability for periodogram peaks was estimated as described by Ruf^33^ and later developed by Baluev^34^. Intrinsic periodicity can be divided in circadian (i.e., regular fluctuations in spontaneous activity with a period of ~24 h) and ultradian (i.e., quasi-regular fluctuations in spontaneous activity with period shorter than 24 h, typically below 12 h). Both circadian and ultradian rhythms are modulated by the SCN^35–38^, but circadian periodicity is typically entrained by environmental stimuli (e.g., light, feeding schedule), while ultradian periodicity is driven rather by instrinsic factors such as cycles in behavioral arousal and episodic glucocorticoid release^39^.

We analyzed the variability of spontaneous activity over 96 h of continuous recording at baseline and after the phase advance by estimating the intra-daily variability (IV), and by means of detrended fluctuation analysis (DFA). IV was calculated as the ratio between the average squared differences between consecutive timebins (pooled activity over 5 min) and the global variance over the entire epoch^40,41^. IV typically ranges between 0 and 2; low IV indicates smooth fluctuations in activity levels; high IV indicates fragmented activity patterns characterized by abrupt transitions between episodes of intense activity and inactive periods^40,41^. For DFA, we ran linear regression analysis for residual variance in time series after detrending against the time scale used for detrending. The intervals for detrending ranged from 20 min to 21.3 h (i.e., in ultradian range) in equally spaced exponential increments. The correlation coefficient of a linear regression in double-logarithmic plot translates into a scaling exponent, and describes the long-term autocorrelation patterns embedded in the time series^42^. Complex time series with fractal-like patterned irregularity yield values between 0.5 and 1: positively correlated fluctuations yield a scaling exponent around 0.5; values around 1 suggest strong underlying regularity (such as diurnal rhythm); values approaching 1.5 indicate unbounded fluctuations resembling random walk (i.e., Brownian noise)^42,43^. The scaling exponent in young, healthy rodents and humans is around 0.8^36,37^.

### Forced-swim test

We evaluated depression-like behavior in mice aged 6 or 12 mo (*N* = 8–12 per treatment) by means of forced-swim test (FST), as described elsewhere^27,44^. Briefly, the animals were individually placed for 6 min in plastic cylinders (24 cm height, 20 cm diameter) filled with water (23.5 °C) to a depth of 16 cm, and videotaped. The footage was analyzed offline by one investigator who was blind to the treatment and exposure conditions. Immobility was defined as continuous passive floating lasting for at least 2 s. After testing, the mice were dried with paper towels and returned to the homecage to recover. The total immobility time over the last 5 min of recording was used for subsequent analyses.

### Protocol for sample collection—perfusion-fixed tissue

The mice were killed by an overdose of anesthetic (sodium pentobarbital, 150 mg/kg) and perfused transcardially with ice-cold buffered saline followed by 4% paraformaldehyde. The brains were removed and postfixed with 4% paraformaldehyde overnight, then cryoprotected with 15% sucrose. The brains were cut along the midline and processed for immunohistochemistry as follows: the right hemisphere (injected with retroviral particles, see below) was embedded in TopVision™ Low Melting point Agarose (5% Thermo Scientific) and cut in 70 μm thick coronal sections with a vibratome (Leica VT1000s) for confocal microscopy imaging. The left hemisphere was quickly frozen on dry ice, and 20 μm thick sections were cut with a cryostat. For stereological counting of DCX + cells, we collected 2–3 slices every 200 μm throughout the hippocampal formation. Between the sections saved for immunohistochemistry we used the trim function of the cryostat to cut 140 μm thick sections for microdissection of hippocampal samples for RNA extraction.

### RNA extraction and gene expression analysis

The mice were killed by an overdose of anesthetic (sodium pentobarbital, 150 mg/kg) and perfused transcardially with ice-cold buffered saline followed by 4% paraformaldehyde. The brains were removed and postfixed with 4% paraformaldehyde overnight, then cryoprotected with 15% sucrose. For gene expression analysis, the SCN was obtained by dissecting a small piece of tissue (~0.5 × 0.5 × 0.5 mm^3^) from the anterior hypothalamus (located on the ventral extent of the brain, dorsal and posterior to the optic chiasm, in the anterior wall of the third ventricle; atlas coordinates bregma −0.84−46 mm, dorsal 0–0.5 mm; lateral 0.5 mm on either side of the midline). The accuracy of SCN sampling was verified by the relative mRNA expression levels for V1a and V1b AVP receptors (V1b expression hardly detectable; see ref. ^45^). In our samples, the mRNA expression of V1b AVP receptors was hardly detectable, indicating negligible contamination with surrounding hypothalamic tissue. The hippocampus was sampled by microdissection from frozen sections (see Supplementary Materials and Methods). RNA extraction was performed using FFPE RNA Purification Kit (Norgen Biotek, Montreal, Canada) according to the manufacturer instruction. The concentration of RNA was measured by NanoDrop 1000 spectrophotometer (Thermo Scientific, Wilmington, DE, USA), and RNA degradation was assessed using an RNA 6000 Nano Kit (Agilent Technologies Inc., Santa Clara, CA, USA) running on an Agilent 2100 Bioanalyzer system (RIN range: 5.1–6.2). The RNA (1 µg template/sample) was reverse-transcribed into cDNA using 0.5 µg of oligo-dT primer according to the instruction of Maxima First Strand cDNA Synthesis kit (Thermo Fisher). Amplification reactions were performed on a QuantStudio 5 thermocycler (Applied Biosystems) using SYBRGreen PCR MasterMix (Thermo Fisher) under the following conditions: initial denaturation 50 °C for 2 min followed by 95 °C for 10 min; followed by 40 amplification cycles (95 °C for 15 s, then annealing temperature (60 °C) for 1 min). The specificity of the qRT-PCR reactions was evaluated by including a dissociation stage to the melting curve analysis. Primer sequences, annealing temperatures, and length of amplification product are available in Supplementary Table. The mRNA expression was normalized against the housekeeping gene glyceraldehyde 3-phosphate dehydrogenase (GAPDH), and the relative regulation was estimated using the 2^−ΔΔCt^ method.

### Analysis of glucocorticoid receptor (GR) activation in the hippocampus

Inactive GR is located in the cytosol in molecular complexes built around a chaperone protein (heat shock protein 90, Hsp90). Upon binding to GC, GR is released from Hsp90 and translocates to the nucleus, where it forms homodimers that bind to the DNA and regulate gene expression^46^. GR complexes in the hippocampus were assessed using in situ proximity ligation assay (PLA)^47,48^ The mice were injected with an overdose of sodium pentobarbital (150 mg/kg i.p.) and exsanguinated by transcardial perfusion with ice-cold PBS, followed by 4% paraformaldehyde. The brains were removed from the skull, postfixed in 4% paraformaldehyde at 4 °C overnight, then cryoprotected using 10% sucrose (0.02% sodium azide) for 3 days, followed by 30% sucrose (0.02% sodium azide) until they sank to the bottom. The brains were snap-frozen in isopentane (−55 °C for 30 s) before sectioning in a cryostat. Brain sections (35 µm thick) through the hippocampal formation were collected at bregma level −3.6 mm and stored in Hoffman solution (250 mL 0.4 M PBS, ethylene glycol 300 mL, 300 g sucrose, 10 g polyvinylpyrrolidone, 9 g NaCl, and high purity water to 1000 mL) at −20 °C until further use. Free-floating PLA was performed using the DUO92101 kit as instructed by the manufacturer. All procedures were performed at room temperature unless specified otherwise. The sections were washed 3 × 5 min in PBS, followed by 20 min of quenching in 10 mM glycine (Sigma-Aldrich), then washed 2 × 5 min in PBS. The sections were then permeabilized with 0.1% Triton X-100 for 30 min before a final washing step of 2 × 5 min in PBS and blocking step of 30 min in blocking buffer (SuperBlock, Thermo Scientific). The sections were incubated with primary antibodies diluted in blocking buffer overnight at 4 °C, with the following antibodies: mouse monoclonal anti-GR (1:100, SAB4800041), rabbit polyclonal anti-GR (1:50, sc-8992) and rabbit polyclonal anti-HSP90 (1:25, SAB4300541). Control experiments included only one primary antibody. The following day the sections were washed 2 × 5 min in blocking buffer and transferred to 37 °C humidified chamber with the probes (1:5, PLA Probes) for 2 h. Afterward the slices were washed 2 × 5 min under gentle agitation first in blocking buffer, then in PBS. Sections were incubated with the hybridization-ligation solution (1:5, Ligation Stock (5×) in dH_2_O, 1:40, Ligase (1 U/uL)) for 1 h in a humidified chamber at 37 °C. The slices were washed twice in Buffer A (8.8 g NaCl, 1.2 g Tris base, 0.5 mL Tween 20 in 1000 mL water adjusted to pH 7.4 with HCl), before incubation with the rolling circle mixture (1:5, Amplification Stock (5×) in dH_2_O, 1:80, Polymerase (10 U/ul)) for 100 min in humidified chamber at 37 °C. The slices were washed 2 × 10 min in Buffer B (5.84 g NaCl, 4.24 g Tris base, 26 g Tris-HCl in 1000 mL water adjusted to pH 7.5 using HCl) shielded from light at RT under gentle agitation, then mounted on microscope slides with mounting medium (Mounting medium with DAPI, Duolink, Sigma-Aldrich), coverslipped, sealed with nailpolish, and stored at −20 °C until imaging. The PLA signal was detected using a Leica TCS-SL confocal microscope (Leica) and quantified with Duolink ImageTool 1.0.1.2 (Sigma-Aldrich). A stack of 20 adjacent optical slices (1 µm thick) was acquired for blue (DAPI) and red (PLA) channels. The PLA signal was visible as intense spots (see Supplementary Fig. S2) located in the cytosol or inside the nucleus. The images used for analysis were generated from the red channel by maximum projection intensity of confocal stacks. We estimated the signal density within manually delineated regions of interest (ROI) inside the pyramidal cell layer of hippocampal CA as the proportion of area occupied by red signal after applying a threshold. A number of 3 images per area for each animal (*N* = 4 animals/group) was used for deriving the average signal density in the hippocampus and was used for subsequent analyses.

### State portraits and analysis of coupling between clock gene expression

Animals were killed within an interval spanning 4.5 h around the expected diurnal trough in Bmal1 expression in the SCN (early subjective night)^49^. The relative mRNA expression for core clock genes Bmal1 and Per1 was normalized to the average of all samples before estimating the fold regulation using the 2^−ΔΔCt^ method. We assessed the coupling between oscillations in core clock gene expression by means of state portraits^50^, where the state of an individual animal (data point) is described either by the expression of Bmal1 and Per1 in the same brain region, or by the expression of Bmal1 in two brain regions (SCN and hippocampus). Synchronized oscillations are defined as oscillations which are phase-locked, i.e., they have the same period, and the time difference between occurrence of peaks is constant. Under steady photic entrainment, when the oscillations in clock gene expression are synchronized^51^, the state portraits approximate an arch of an ellipse, where the order of positions of datapoints follows the temporal sequence of sample collection. First we assessed the synchronization of clock gene expression within brain region by plotting the relative expression level of Per1 against Bmal1 mRNA in SCN and hippocampus, respectively. Second we assessed the synchronization between the oscillations in Bmal1 expression across regions by plotting the relative expression level of Bmal1 mRNA in hippocampus against the relative expression level in the SCN.

### Analysis of clock gene expression in skin fibroblasts

Tissue samples (~0.25 cm^2^) were harvested from the ear of adult (6 mo) control and DEX-exposed mice under terminal anesthesia. The tissue was rinsed in Hank’s Balanced Salt Solution (HBSS) (Life Technologies Europe BV, Stockholm, Sweden), then minced with sterile razor blade into Collagenase (Type XI-S) (Sigma-Aldrich, Sweden) (30 min at 37 °C). After digestion, 3 mL of DMEM Medium (Life Technologies) supplemented with 10% Fetal Bovine Serum and 1% Penicillin/Streptomycin (Life Technologies) was added to a 6 cm plate and the samples were incubated at 37 °C for at least 6 days. After passaging (0.05% Trypsin-EDTA; Invitrogen), the cells were plated in 35 mm dishes in MEF medium (DMEM Medium +10% FBS +1% pen/strep) at a density of at least 50 k/cm^2^. After 24 h, the expression of clock genes was synchronized by exposing the fibroblasts to 1 µM DEX. The cells were collected between 36 and 63 h after synchronization. The relative expression of Bmal1 was assessed by qPCR with GAPDH as housekeeping gene. Circadian oscillations in clock gene expression were analyzed by means of cosinor rhythmometry^52,53^.

### Analysis of dendritic arborization

The maturation of newborn granule neurons was investigated in cells expressing green fluorescent protein (GFP) delivered using a retroviral vector injected stereotactically in the dorsal hippocampus as described earlier^44^. The injection site was located as follows, in relation to bregma: antero-posterior, −2.6 mm; medio-lateral, +1.75 mm; dorso-ventral, −2.0 mm (from dura)^54^. The GFP-expressing cells were analyzed 4 weeks after the injection, which corresponds to the full morphological maturation of the retrovirus labeled granule cells (21–28 days post infection)^55^. For the morphological analysis, all brain sections from the hemisphere injected with retroviral particles through the entire hippocampus (12–15 sections/animal) were collected in PBS for 10 min washing, and subsequently processed for free-floating immunohistochemistry as described above. Sections were then mounted on Microscope Slides cut edges frosted (VWR International) with Fluorescent Mounting Media (DAKO). We used rabbit anti-mouse GFP (1:1000, Thermo Fisher; USA) and Pierce^TM^ Donkey anti-Rabbit IgG (H + L) Cross Adsorbed Secondary Antibody 543 (1:200, Thermo Fisher; USA). An average optical thickness of 50 μm was imaged (from the slice thickness of 70 μm). Z-series at 1 μm intervals were acquired with a Plan-Apochromat 20 × 0.75 objective, digital zoom 1.5, on a Zeiss ZEN 2009 LSM 510 META confocal system. A total of 5–12 cells from each mouse were analyzed for each data point. All images were analyzed in FiJi^56^ using a semiautomatic procedure. Images of dendritic arborization were deconvolved with Iterative deconvolve 3D plugin and manually traced with Simple Neurite Tracer plugin before running automated Sholl analysis. Granule cells displaying truncated dendrites, or grossly departing from the plane of the coronal sections, were excluded from the analysis. The number of neurons analyzed ranged between 4 and 12/animal (*N* = 7–12 animals/treatment). See also ref. ^44^ for further methodological details.

### Analysis of neurogenesis

Neurogenesis and the maturation of newborn neurons were analyzed as previously described^44^ in mice aged 6 or 12 mo. Briefly, proliferating of neuronal progenitors were labeled by 5-ethynyl-2 deoxyuridine (EdU) uptake. EdU (50 mg/kg/day) was injected i.p. for 5 consecutive days before killing the animals at 6 months. The EdU uptake was visualized using the Click-iT™ EdU Alexa Flour™ 488 Imaging kit (Thermo Fisher) as instructed by the manufacturer. Postmitotic progenitors committed to the neuronal lineage were identified in 12 mo animals by immunohistochemical detection of DCX expression. The number of EdU or DCX-positive cells in the subgranular zone was assessed on 20 μm thick frozen coronal section collected every 200 μm from the left hemisphere (i.e., not injected with viral particles). The slices were mounted on SuperFrostPlus™ Microscope Slides (VWR International) and circled with Dako pen. After 10 min re-hydration with phosphate buffered saline (PBS), sections were treated with blocking mix (PBS, 5% Normal Donkey Serum, 0.3% Triton-X) for 2 h. Sections were then incubated overnight at 4 °C with primary antibody. After washing with PBS 3 × 5 min, sections were incubated with secondary antibody for 2 h at room temperature. After washing 2 × 5 min with PBS, sections were counterstained with 4′,6′-diamidino-2-phenylindole (DAPI, 1:1000, Sigma-Aldrich) for 5 min, washed with PBS for 3 × 5 min and mounted with coverslips. The primary antibody used: Guinea pig anti-mouse DCX antibody (1:1000, Millipore; CA). The secondary antibody used was Donkey anti-Guinea pig 488 (1:200, Alexa). The DCX-positive cells in the granule cell layer of the DG were counted throughout the entire hippocampal structure in a stereological design on equally spaced 20 μm sections (200 μm between consecutive slides). The total number of cells was estimated by multiplying the number of counted cells with the inverse sampling fraction.

### Statistical analysis

All statistical analyses were performed in Statistica™ 13 (TIBCO Software Inc., Palo Alto, CA, USA). The onset of active phase and the variability in spontaneous activity were analyzed using a mixed model ANOVA with repeated measures, followed by Dunnett’s *post hoc* test against DEX as reference group. mRNA expression was analyzed first by one-sample *t*-test vs. “no-regulation” value (=1), then between-group comparisons were analyzed by two-sample *t*-test. The time to re-entrain, immobility time in FST, and PLA group differences were analyzed using simple ANOVA models followed by Dunnett’s *post hoc* test against DEX as reference group. The differences between Sholl curves were reported only when pointwise differences (*t*-test) were found significant for at least three consecutive points (see also ref. ^44^). Differences are reported as significant if *p* < 0.05. All individual comparisons are one-sided. The data are shown as average ± SEM.

## Results

### DMI treatment restores circadian entrainment of spontaneous activity in DEX mice

**Fig. 1:**
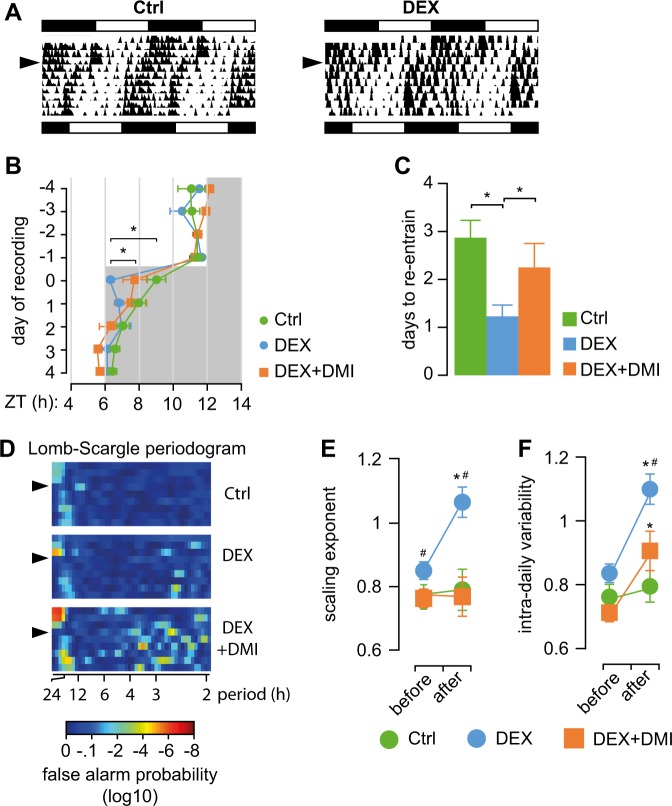
Circadian re-entrainment after a 6 h advance in the onset of dark phase. **a** Illustrative double-plotted actograms for controls (Ctrl) and mice exposed to DEX in utero (DEX). The LD cycle before and after the phase advance is depicted by the black and white bars at the top and the bottom, respectively; arrowhead indicates the time of phase advance. **b** Active phase onset in relation to the LD cycle. Note that DEX mice re-entrain to the shifted LD cycle virtually without delay, while DMI treatment delays entrainment. *p < 0.05, mixed model ANOVA with repeated measures, followed by contrast analysis. **c** Quantification of number of days it takes for spontaneous activity to entrain to the shifted LD cycle. *p < 0.05, one-way ANOVA followed by Dunnett’s *post hoc* test vs. DEX. **d** Lomb-Scargle periodogram illustrating the effect of abrupt phase advance on the periodicity of spontaneous activity. Arrowhead indicates the time of phase advance. Circadian periodicity is lost for about three LD cycles in DEX mice, while in controls it is largely preserved throughout the experiment. DMI-treated DEX mice display robust circadian rhythmicity. **e**, **f** Analysis of variability in spontaneous activity. **e** The scaling exponent describes the scale-dependence of variability in ultradian range. Higher scaling exponent in DEX mice suggest more rigid patterns of activity, while values above 1 indicate unbounded fluctuations approaching random walk. **f** Intra-daily variability estimates variability in activity, and is an intrinsic feature of the regulation of spontaneous activity at ultradian scale. *p < 0.05 “after” vs. “before”; ^#^*p* < 0.05 vs. Ctrl; mixed model ANOVA with repeated measures followed by Dunnett's *post hoc* test

We investigated the circadian entrainment of spontaneous activity in response to a 6-h phase advance in the onset of dark (Fig. 1a). Given that spontaneous activity is regulated by the SCN, the expected response should be a gradual advance in onset of activity to match the LD cycle and presumably negligible alterations in intrinsic rhythmicity^36,38^. In control mice, we found that re-entrainment was completed after ~3 days (Fig. 1b, c), while the intrinsic periodicity (Fig. 1d) and variability (Fig. 1e, f) were not significantly altered by the abrupt advance of the onset of dark phase. In contrast, in DEX-exposed mice we found that re-entrained of spontaneous activity occurred without delay (Fig. 1b, c). In addition, circadian periodicity was lost for ~3 LD cycles following the phase shift in DEX mice (Fig. 1d). We then investigated the scale-dependence of variability in locomotor activity in ultradian range by means of DFA, and found that the scaling exponent in DEX-exposed mice was higher under steady entrainment, and significantly increases following the phase advance (Fig. 1e). Similarly, the intra-daily variability increased in DEX-exposed mice following the phase advance (Fig. 1f). DMI-treated DEX mice displayed a significant delay in re-entrainment (Fig. 1b, c), and the circadian periodicity was restored immediately after the phase shift, similar to controls (Fig. 1d). In addition, DMI treatment attenuated the effects of phase advance on variability in spontaneous activity in DEX mice (Fig. 1e, f).

**Fig. 2:**
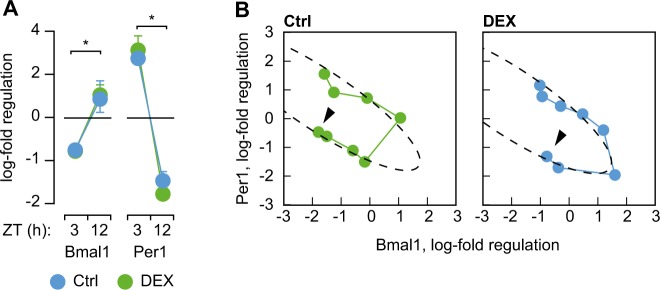
Photic entrainment of SCN. **a** Clock gene expression in the SCN of DEX mice display fluctuations between diurnal peak and trough similar to controls. *p < 0.05, paired student’s *t*-test. **b** State portraits illustrating coupled oscillations in clock gene expression synchronized with the LD cycle. Connected datapoints indicate the temporal sequence of sample collection (first sample indicated by arrowhead)

The alterations in circadian entrainment and rhythmicity we found in DEX-exposed mice can be due to impaired photic entrainment of the SCN, or to uncoupling between peripheral oscillators and the central clock. To address this question we first analyzed the expression of clock genes in the SCN in mice maintained under steady photic entrainment. We found that DEX mice display robust variations between the expected peak and trough of expression, similar to controls (Fig. 2a), indicating that photic entrainment of the SCN was not altered by prenatal exposure to DEX. Similarly, the state portraits of clock gene expression in the SCN indicate that mRNA expression of Per1 and Bmal1 are synchronized with the LD cycle in both controls and DEX mice (Fig. 2b). Therefore we hypothesized that the phenotype of DEX mice is the result of altered coupling between SCN and peripheral oscillators.

### Treatment with DMI restores the coupling of SCN with peripheral oscillators

**Fig. 3:**
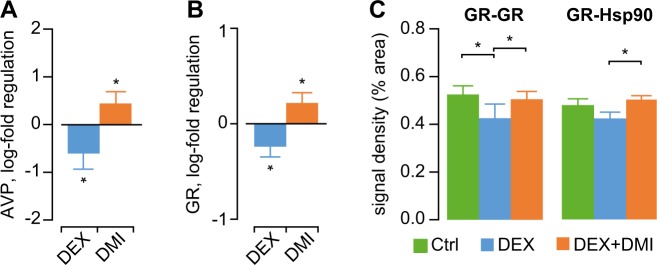
Alterations in mechanisms of coupling between peripheral clocks and the SCN. **a** mRNA expression for AVP in SCN is down-regulated in DEX mice, but is upregulated following DMI treatment. *p < 0.05, single sample *t*-test vs. log-fold regulation = 0 (“no regulation”). **b** Hippocampal mRNA expression of GR in DEX mice is down-regulated as compared with controls, and is upregulated by DMI treatment. *p < 0.05, single sample *t*-test vs. log-fold regulation = 0 (“no regulation”). **c** Assessment of GR activation by means of PLA. The PLA signal density (% from ROI area) for activated GR is decreased in DEX mice as compared with controls. DMI treatment increases the signal for both inactive (GR-Hsp90) and activated (GR-GR) fractions of GR in DEX mice. *p < 0.05, one-way ANOVA followed by Dunnett’s *post hoc* test vs. DEX

AVP is the main molecular output of SCN involved in the synchronization of peripheral oscillators with the central clock^19,57,58^. We analyzed the mRNA expression for AVP in the SCN at the expected diurnal peak of expression (early subjective night) and found significant downregulation in DEX mice (Fig. 3a), suggesting weaker peak output. AVP mRNA expression was upregulated after DMI treatment in DEX mice (Fig. 3a). The coupling of peripheral oscillators with the SCN relies on GC signaling^59,60^, and the hippocampus plays a critical role in the regulation of HPA axis^61,62^. Therefore we measured the hippocampal mRNA expression of GR and found it was downregulated in DEX mice as compared with controls, but was upregulated after DMI treatment (Fig. 3b).

Next we asked whether the differential gene expression regulation was accompanied by changes in GR activation. We measured in situ PLA signal intensity for activated (GR-GR homodimers) and inactive (GR-Hsp90 heterodimers) fractions of GR in the Cornu Ammonis (CA) region of the hippocampus. The PLA signal for activated GR was localized within the nucleus (Supplementary Fig. S2A), while the inactive form was localized primarily in the cytosol (Supplementary Fig. S2B). The signal for activated GR was lower in the DEX-exposed mice than in controls, while the signal for inactive GR was not different between groups (Fig. 3c). After DMI treatment, the PLA signal for both activated and inactive GR was increased in DEX-exposed mice (Fig. 3c).

**Fig. 4:**
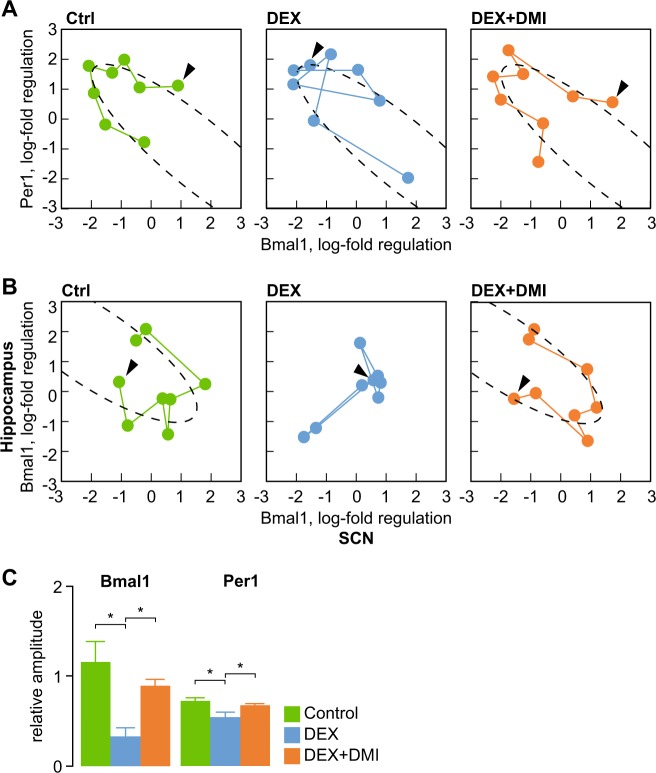
DMI treatment restores coupling between SCN and peripheral clocks. **a** State portraits assessing the intrinsic clock mechanism and the synchronization with the LD cycle in hippocampal samples. The mapping of datapoints approximates an ellipse and are consistent with the order of sampling in control animals. In DEX mice, the mapping approximates an ellipse, but is not consistent with the temporal order of sample collection, suggesting that the intrinsic clock mechanism is preserved, but is not synchronized with the LD cycle. The correspondence with the temporal sequence is recovered after DMI treatment. **b** State portraits assessing the coupling between oscillations in clock gene expression between SCN and hippocampus. DEX mice do not display signs of coupling, while the pattern following DMI treatment is undistinguishable from controls. Connected datapoints indicate temporal sequence of sample collection (first sample indicated by arrowhead). **c** Amplitude of oscillations in clock gene expression in primary skin fibroblasts. *p < 0.05, one-way ANOVA followed by Dunnett’s post hoc test vs. DEX

We next addressed the question of desynchronization between SCN and peripheral oscillators and the restoration of coupling following DMI treatment. First, we assessed the intrinsic clock function in the hippocampus and analyzed the state portraits for Bmal1 and Per1 expression (Fig. 4a). As expected, the state portrait for control animals closely followed the expected ellipse contour, and was consistent with the order of sample collection (see also ref. ^63^). In contrast, the relationship between the expression of Bmal1 and Per1 approximated and ellipse segment in DEX mice, but the order of mapped points appeared shuffled in relation to the temporal order of sample collection (Fig. 4a). In DMI-treated DEX mice, the order of datapoints matched the temporal order of sample collection, similar to controls. This suggests that the intrinsic clock mechanism may be preserved, but the synchronization with the LD cycle was lost in DEX mice. To test this hypothesis, we plotted the relative level of mRNA expression for Bmal1 in the hippocampus against the relative expression level of Bmal1 mRNA expression in the SCN (Fig. 4b). The state portrait for control mice is consistent with coupled oscillations in Bmal1 expression between hippocampus and SCN, as described previously^64^. In contrast, we found no evidence of coupling between Bmal1 expression in SCN and hippocampus in DEX mice (Fig. 4b). Following treatment with DMI, the state portraits indicate that the oscillations in hippocampal clock gene expression is synchronized between hippocampus and SCN, as seen in control animals (Fig. 4b).

To investigate the response of peripheral oscillators to entrainment by GR-mediated signaling we analyzed clock gene expression in primary skin fibroblasts following synchronization by GR activation (see also ref. ^27^). The amplitude of oscillations in core clock genes Bmal1 and Per1 was dampened in cells isolated from DEX mice. In contrast, skin fibroblasts isolated from DMI-treated DEX mice display increased amplitude of oscillations in clock gene expression as compared with cells isolated from untreated mice (Fig. 4c).

### Treatment with DMI at 6 months prevents the onset of depression-like phenotype at 12 months

**Fig. 5:**
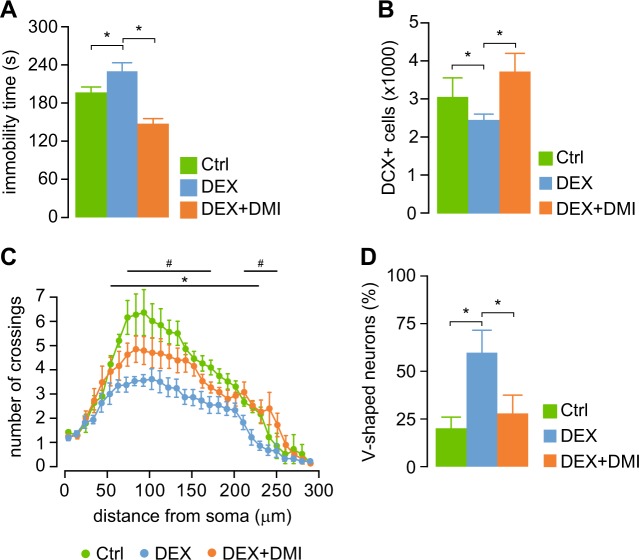
DMI treatment in DEX mice at 6 mo prevents the onset of depression-like phenotype at 12 mo. **a** Immobility time in FST at the age of 12 mo is lower in DEX mice treated with DMI at 6 mo than in untreated DEX mice. *p < 0.05, one-way ANOVA followed by Dunnett’s *post hoc* test vs. DEX. **b** The number of newly generated neurons in the hippocampal dentate gyrus (DCX + cells) is lower in DEX mice as compared with controls, while DMI treatment at 6 mo prevented the decrease in hippocampal neurogenesis. **c**, **d** The alterations in morphology of newly generated hippocampal neurons in DEX mice aged 12 mo are prevented by DMI treatment at 6 mo. **c** The pattern of dendritic arborization of newly generated neurons in DEX miced aged 12 mo which were treated with DMI at 6 mo is not different from controls. The number of crossings is decreased globally in DEX mice, while in DMI-treated DEX mice the number of crossings in proximal and distal regions is significantly increased as compared with untreated DEX mice. *p < 0.05, student’s *t*-test DEX vs. Ctrl. ^#^*p* < 0.05 student’s *t*-test DEX + DMI vs. DEX. Significantly different points shown only if the diference is significant for at least three consecutive samples. **d** The number of V-shaped, abnormal neurons is reduced in DEX mice by prior treatment with DMI. *p < 0.05, one-way ANOVA followed by Dunnett’s *post hoc* test vs. DEX

Lastly, we asked whether DMI treatment affects the onset of depression-like behavior in DEX mice. At the age of 6 mo, DEX mice did not display increased immobility time in FST, and hippocampal neurogenesis and neuronal morphology were not different from controls (Supplementary Fig. S3). When DEX mice were tested at the age of 12 mo, the immobility time in FST was significantly lower in the DEX mice treated with DMI at 6 mo than in untreated DEX mice (Fig. 5a). In addition, neither hippocampal neurogenesis (Fig. 5b), nor the morphology of adult-born neurons (Fig. 5c, d) were altered in 12-mo-old DEX mice treated with DMI at 6 mo.

## Discussion

Here we show that the alterations in photic entrainment of spontaneous activity in mice exposed to DEX in utero are associated with weaker entrainment of peripheral clocks. Treatment with the antidepressant DMI restores photic entrainment of spontaneous activity. A possible mechanism is the restoration of coupling between SCN and peripheral clocks by enhancing GR-mediated signaling. In addition, mice exposed to DEX in utero and treated with DMI at 6 months of age do not display alterations in hippocampal neurogenesis and increased immobility time in FST at the age of 12 months.

Spontaneous activity behaves like a peripheral clock and is regulated by the SCN at both circadian and ultradian timescales^36,38,65^. Therefore, re-entrainment after a sudden shift in the LD cycle should occur gradually, while rhythmicity and variability in spontaneous activity are preserved, as observed in control mice. In contrast, in DEX mice, spontaneous activity appears to re-entrain without delay and both periodicity and variability are profoundly disrupted. Accelerated re-entrainment has been described in experimental models where the coupling between SCN and peripheral clocks was impaired by knocking out the expression of AVP receptors in the SCN^57,66^, or by disrupting GC signaling^20,22,23^. Disruption of AVP signaling within the SCN renders circadian timekeeping more sensitive to perturbations by weakening the coupling between neurons within the SCN shell, which in turn weakens the entrainment of peripheral clocks^58,66–68^. Clock gene expression in SCN is intrinsically rhythmic and subject to photic entrainment via the RHT^69,70^, while circulating GC are required for SCN to entrain downstream oscillators^20,23,60^. Clamping circadian oscillations in GC secretion uncouples peripheral clocks from the SCN and facilitates the re-entrainment of spontaneous activity^20,22,23^. Therefore, the patterns of alterations following the phase advance in subjective night onset suggests altered coupling between SCN and peripheral clocks in DEX mice. A higher scaling exponent in DEX mice at baseline (i.e., constant LD cycle) is in agreement with our previous report^27^. The increase in scaling exponent above 1 after the phase advance is particularly relevant, since it indicates random fluctuations in activity^43^, probably reflecting a transiently aggravated uncoupling from SCN regulation, as previously described in AVP-receptor knockout mice^66^.

The synchronization of clock gene expression in the SCN with the LD cycle indicates that photic entrainment of the SCN is preserved in DEX-exposed mice. At the same time, the desynchronization between hippocampal clock gene expression and the LD cycle suggests uncoupling between central and peripheral clocks. We attempted to restore the coupling between SCN and peripheral clocks by treating DEX mice with DMI because (1) increasing the availability of norepinephrine (NE) has been shown to upregulate AVP expression in the SCN^71–73^; and (2) DMI potentiates GR-mediated signaling^29–31^. Indeed, we found that the mRNA expression of AVP in the SCN was upregulated after DMI treatment in DEX mice, which presumably strengthened circadian output from the SCN. In line with earlier reports^74–76^, DMI upregulated hippocampal mRNA expression of GR, which was accompanied by increase in both inactive (cytosolic) and activated (nuclear) fractions of GR, as shown by the increase in PLA signal. This reinforced the negative feedback loop required for the regulation of pulsatile secretion of GC (see also ref. ^32^), thereby facilitating the entrainment of peripheral clocks. As a consequence, intrinsic rhythmicity was stabilized and re-entrainment of spontaneous activity delayed in DMI-treated DEX mice. Further support is provided by the synchronization of oscillations in hippocampal clock gene expression with the LD cycle, which indicates that hippocampal clock machinery is entrained by the SCN. In addition, we have used an ex vivo preparation (primary cultures of skin fibroblasts) to assess the response to entrainment in a peripheral clock. The amplitude of synchronized oscillations in clock gene expression was lower in DEX mice than in controls, in agreement with our earlier report^27^, but was restored in mice DEX mice treated with DMI, indicating a more robust entrainment by a pulse of GR agonist.

Excess GC signaling is a mechanism suggested to underlie the long-term effects of prenatal adversity and IUGR^77^. In human populations, IUGR is associated with increased risk of depression, particularly in men^78,79^, and the reprogramming of the SCN has been suggested to have a relevant contribution^80^. Mouse pups exposed to DEX in utero have lower body weight at birth, and we have previously reported alterations in photic entrainment of spontaneous activity in males only^27^. Alteration of photic entrainment specifically have been described following lesions in the NE system^81^. The locus coeruleus-NE system, which sends widespread projections in the forebrain and plays a major role in regulating arousal and the sleep-wake cycle^81,82^, is particularly relevant in relation to the mechanism of action of DMI. In this study we have treated DEX mice with DMI at the age of 6 months in order to reverse the alterations in photic entrainment. Coupling between oscillators is a critical determinant of the ability to be entrained by external pacemakers^83^. Our data suggest that DMI treatment restored the coupling between peripheral clocks and the SCN, which may account for the restoration of circadian entrainment of spontaneous activity. Hippocampal neurogenesis is regulated by clock genes and fluctuations in circulating GC^84–86^, and the restoration of circadian entrainment has been suggested to underlie the effects of specific antidepressants (e.g., ketamine^87,88^, agomelatine^89^). This may explain the fact that, treatment with DMI (which restores circadian entrainment of peripheral clocks), but not with fluoxetine (which requires robust GC oscillation for antidepressant effects^90^), restored hippocampal neurogenesis in our model^27,44^. The preservation of hippocampal neurogenesis in 12-mo-old DEX mice treated with DMI at 6 mo suggests that DMI treatment favors a robust circadian entrainment of peripheral clocks for long enough to prevent the onset of DEX-induced alterations in hippocampal neurogenesis and depression-like behavior. Taken together, our data indicate that DMI treatment restores the synchronization of peripheral clocks with the SCN and support the hypothesis that altered circadian entrainment of activity is a modifiable risk factor for depression onset.

## Supplementary information

Supplementary Information

## Data Availability

All Matlab™ code used for generating data is available upon request.
