# Supplementary material for: Desipramine restores the alterations in circadian entrainment induced by prenatal exposure to glucocorticoids
Source: Transl Psychiatry. 2019 Oct 17;9:263. doi: 10.1038/s41398-019-0594-3 (PMC6797805; doi:10.1038/s41398-019-0594-3)
Supplement: Supplementary file 1 — Supplementary Information [file 41398_2019_594_MOESM1_ESM.docx]

**Desipramine restores the alterations in circadian entrainment induced by prenatal exposure to glucocorticoids**

**Running title:** DMI restores circadian entrainment in DEX-exposed mice

**Authors:** Stefan Spulber^*^; Mirko Conti^*^; Frederik Elberling; Marilena Raciti; Dasiel Oscar Borroto-Escuela; Kjell Fuxe; Sandra Ceccatelli.

Department of Neuroscience, Karolinska Institutet, Stockholm, Sweden

^*^ - shared first authorship

**Contents:**

**Supplementary Figure S1 –** Timeline of experimental procedures

**Supplementary Figure S2 –** PLA signal for inactive and activated GR

**Supplementary Figure S3 –** Depression-like phenotype at the age of 6 mo

**Supplementary Table –** Primer sequences, annealing temperatures and amplicon lengths


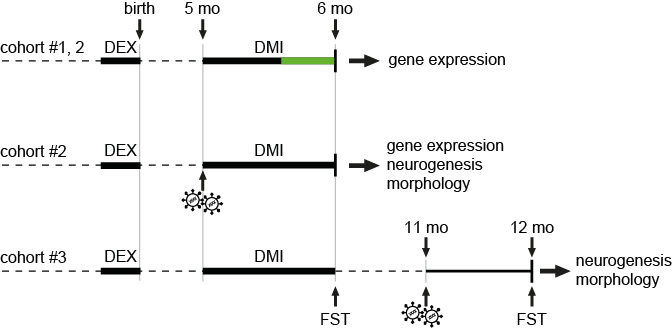
**Supplementary Figures**

**Supplementary Figure S1.** Graphical depiction of experimental timelines for different cohorts. Time of monitoring spontaneous activity on TraffiCage™ system outlined in green. Time of sacrifice indicated by vertical lines. The mice were allowed to survive for 4 weeks after the inoculation of viral particles (see also (Conti, 2017; Conti *et al*, 2017) for detailed description of the method). Mice from cohort #2 were used for both recording of spontaneous activity and gene expression, and for neurogenesis and morphology of newly generated neurons. In both experiments we included 4 mice/group originating in different litters.


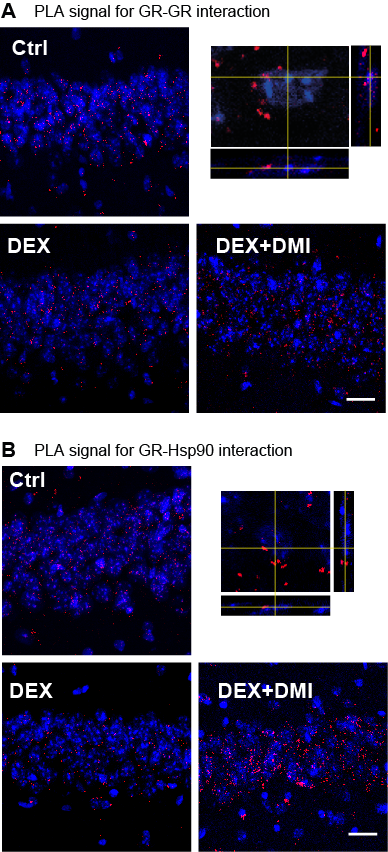
**Supplementary Fig. S2** Illustration of PLA signal for detection of GR-GR (A) and GR-Hsp90 (B) protein interactions. Inserts show the typical pattern of localization of PLA signal in orthogonal view of Z-stacks: (A) inside the nucleus; (B) in the cytosol. The quantification of the PLA signal is shown in Fig. 3C in the main text. Scalebar: 25 µm.


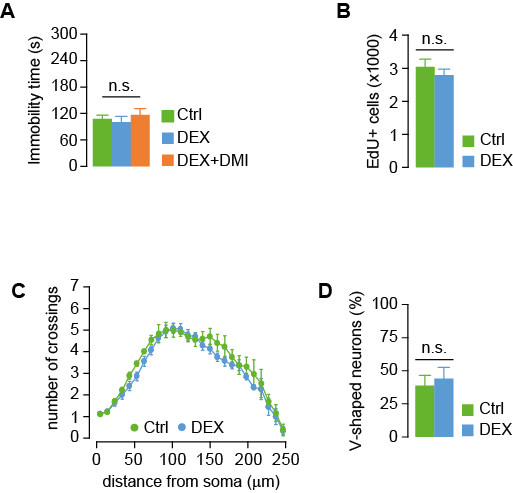
 **Supplementary Fig. S3** Depression-like phenotype at the age of 6 mo (A) Immobility time in forced swim test. Mice exposed to DEX do not display depression-like behavior, and DMI treatment does not have significant effects on immobility time. (B-D) Analysis of neurogenesis and morphology of newly-generated neurons in the dentate gyrus. No alterations in either number (B), or morphology (C, D) of newly-generated neurons is detected in DEX-exposed mice. n.s. – not significant

**Supplementary Table**

| primer | sequence | annealing temperature | amplicon length |
| --- | --- | --- | --- |
| GAPDH | fw CAAGGCCGAGAATGGGAAG  rv GGCCTCACCCCATTTGATGT | 58-60ºC | 91 bp |
| Bmal1 | fw AACCTTCCCGCAGCTAACAG  rv AGTCCTCTTTGGGCCACCTT | 60ºC | 79 bp |
| Per1 | fw CCAGATTGGTGGAGGTTACTGAG  rv GCGAGAGTCTTCTTGGAGCAGTA | 60ºC | 92 bp |
| AVP | fw CGAGTGCCACGACGGTTTTT  rv GCGATGGCTCAGTAGACCC | 60ºC | 162 bp |
| GR | fw CAAGTGATTGCCGCAGTGAA  rv CATCCAGGTGTAAGTTTCTGAAT | 58ºC | 64 bp |
